# The associations of positive and negative mental well-being with physical activity during the COVID-19 across late adulthood

**DOI:** 10.1186/s12889-024-20803-3

**Published:** 2024-11-26

**Authors:** Tiina Savikangas, Tiia Kekäläinen, Anna Tirkkonen, Sarianna Sipilä, Katja Kokko

**Affiliations:** 1 Gerontology Research Center, Faculty of Sport and Health Sciences, University of Jyväskylä, PO Box 35 (viv256), Jyväskylä, FIN-40014 Finland; 2 Faculty of Sport and Health Sciences, University of Jyväskylä, Jyväskylä, Finland

**Keywords:** Physical activity, Mental well-being, Positive affect, Negative affect, Depressive symptoms, Older adults, COVID-19

## Abstract

**Background:**

Mental well-being (MWB) may play an important role in physical activity (PA) behavior, but the independent associations of affective MWB indicators and depressive symptoms with PA are sparsely investigated in late adulthood. We investigated the associations of positive affect, negative affect, and depressive symptoms with PA during the COVID-19 restrictions across late adulthood.

**Methods:**

Cross-sectional data came from two Finnish population-based studies. The younger cohort (*N* = 162, 56% women, 60–61 years) was drawn from the TRAILS study, and the older (*N* = 272, 60% women, 72–88 years) from the PASSWORD study. Data on PA frequency (1–7; from “not at all” to “approximately daily”) and perceived changes in PA (no change/increased/reduced) during COVID-19 restrictions were self-reported. MWB was assessed by positive and negative affect (International Positive and Negative Affect Schedule Short Form) and depressive symptoms (younger cohort: General Behavioral Inventory; older cohort: Geriatric Depression Scale). Associations between MWB and PA were analyzed using linear and multinominal logistic regression models.

**Results:**

When each MWB indicator was investigated separately, higher positive affect was associated with higher PA frequency in both cohorts and with a higher likelihood of increased PA in the younger cohort; higher negative affect was associated with a higher likelihood of reduced PA in the older cohort; higher depressive symptoms were associated with lower PA frequency in both cohorts and with a higher likelihood of reduced PA in the older cohort (*p* ≤ 0.009 for all), In the final models including all three MWB indicators, socioeconomic, and health-related covariates, only the association between higher positive affect and higher odds of increased PA remained significant in the younger cohort (OR = 4.28, *p* = 0.006). In the older cohort, only the associations of higher depressive symptoms with lower PA frequency (β=-0.097, *p* = 0.024) and higher odds of reduced PA (OR = 1.26, *p* = 0.010) remained significant.

**Conclusions:**

Positive affect was independently associated with increased PA during the COVID-19 restrictions in sixty-year-olds. Higher depressive symptoms were independently associated with lower PA frequency and a higher likelihood of reduced PA in 72-88-year-olds. Positive and negative aspects of MWB have different relations to PA among people aged 60 and 70 + when one’s daily routines are restricted.

**Trial registration number:**

ISRCTN52388040 (the PASSWORD study).

**Supplementary Information:**

The online version contains supplementary material available at 10.1186/s12889-024-20803-3.

## Background

Mental well-being (MWB) has a multidimensional structure including both positive and negative aspects [1]. Affective MWB refers to the tendency to experience pleasant and unpleasant feelings, that is, positive and negative affect [2–5]. These aspects are correlated but still distinct [5]. While the benefits of physical activity (PA) for mental health, especially depression, are well-known [6, 7], the role of MWB as an antecedent of PA has received less attention [8]. High positive MWB may serve as a resource for being physically active, whereas high negative MWB may act as a barrier to PA [2, 8–10]. The role of MWB may be of special importance for health behavior when one’s daily routines are compromised, such as during the outbreak of the COVID-19 pandemic.

In the spring of 2020, the breakthrough of the novel COVID-19 disease led to various degrees of social distancing restrictions and the closure of societies worldwide. In Finland, no curfew was declared, but significant restrictions were imposed. For example, people were instructed to work from home, if possible, cafes, restaurants, and public sports facilities were closed, group activities ceased, and gatherings of more than ten people were prohibited. In addition, people aged 70 years and older were obligated to stay in self-quarantine and avoid physical contact with others. Restrictions were gradually loosened after a few months. These measures restricted possibilities to maintain habitual activities but did not prevent PA altogether, especially in people under age 70. Worldwide, PA levels decreased during the restrictions [11], but several studies indicate that a majority of Finnish people in late adulthood, that is, people over the age of 60 years [12], maintained or even increased their PA [13–15].

Individual differences in MWB may be one explanation for why some people were able to maintain a physically active lifestyle during the COVID-19 restrictions, while others withdrew from being active [16]. A few previous studies have indicated that MWB in general was linked to PA during the COVID-19 pandemic [16–19], but affective MWB may be of special importance for the ability to maintain a physically active lifestyle during exceptional circumstances restricting one’s habitual activities. A person with higher affective MWB may find new ways to be physically active since positive affect is linked to approach motivation and goal-directed behavior and negative affect to withdrawal and avoidance [20]. That is, higher positive affect may have facilitated positive adoption of one’s PA routines to overcome the barriers caused by the social distancing restrictions, whereas higher negative affect may have facilitated withdrawal from going out and being physically active. Although high affective MWB is suggested to promote PA in normal circumstances [2, 21–23], research on the relationships between affective MWB and PA during the COVID-19 restrictions is lacking, especially among older adults. Furthermore, the sparse literature is generally limited by convenience sampling and online questionnaires, which may not necessarily reach older age groups and may lead to sampling bias.

A further limitation of the existing literature is that positive and negative affect have been investigated in isolation from depressive symptoms. Positive and negative affect are relatively independent of each other but more strongly related to the experience of depressive symptoms [5]. Depression is typically characterized by depressed mood and loss of energy and interests, that is, high negative and low positive affect, but may also comprise other symptoms such as impaired cognitive function and loss of appetite [5, 24]. To understand the independent associations of positive affect, negative affect, and depressive symptoms with PA, these should be investigated together. This is of special importance in late adulthood, when the mutual relationships of different aspects of MWB may change. In general, late adulthood is characterized by a high level of MWB [12]. Negative affect decreases across adulthood, while positive affect remains relatively stable until very old age [25]. During the COVID-19 restrictions, higher age was associated with fewer depressive symptoms [26, 27], whereas the evidence on the relationship of age with positive and negative affect is mixed. Some studies indicate a positive association [26, 28], others a negative association [29], or no association [30] between affective MWB and age during the COVID-19 restrictions. The discrepancies may be due to different restrictions for different population groups in different countries. While different restrictions applied to people in their sixties and those over 70 in Finland, the relationship between affective MWB and PA may have been different in different phases of late adulthood.

Therefore, this cross-sectional study aimed to investigate the associations of positive affect, negative affect, and depressive symptoms with the self-reported frequency of and changes in PA during COVID-19 restrictions across late adulthood. Based on the previous literature, we hypothesize that positive affect was positively, whereas negative affect and depressive symptoms were negatively associated with PA behavior during the COVID-19 restrictions when investigated separately. This study adds to the existing literature new information about the independent relationships of positive affect, negative affect, and depressive symptoms with PA behavior during circumstances restricting one’s habitual activities by also investigating the associations of the three MWB indicators with PA in relation to each other. To overcome the selection bias related to convenience sampling and online questionnaires in the existing literature, we recruited participants from two ongoing larger, population-based studies representing different phases of late adulthood from 60 to 88 years.

## Materials and methods

### Study designs and participants

This study is based on cross-sectional analyses of data collected in two larger population-based research projects conducted at the Gerontology Research Center and the Faculty of Sport and Health Sciences, University of Jyväskylä, Finland. A total of 434 participants, aged 60–88 years were included.

Data for the younger cohort representing people at the beginning of late adulthood was drawn from the TRAILS study, which is the latest data collection phase of the ongoing Jyväskylä Longitudinal Study of Personality and Social Development (JYLS) [31, 32]. The participants were 60 to 61 years old and represented the Finnish age cohort born in 1959 [32]. Initially, 12 complete second-grade classes were randomly selected from the Jyväskylä area in Central Finland without initial attrition, and the participants have been followed since they were eight years old (initial *N* = 369) [31]. The surviving participants who had not withdrawn from the study and whose address was available (*N* = 301) were contacted in February 2020, when an invitation letter and the Life Situation Questionnaire were mailed to them [32]. The present study sample comprises the participants who attended online or face-to-face interviews, which included PA and MWB questionnaires, between April 2020 and July 2021, i.e., after the outbreak of COVID-19 (*N* = 162, 44% of the initial sample and 54% of the available sample of 301). According to previously published attrition analyses, women were overrepresented in this data collection wave compared to the initial study sample, but the sample was still well representative of their age cohort in Finland [32].

**Fig. 1:**
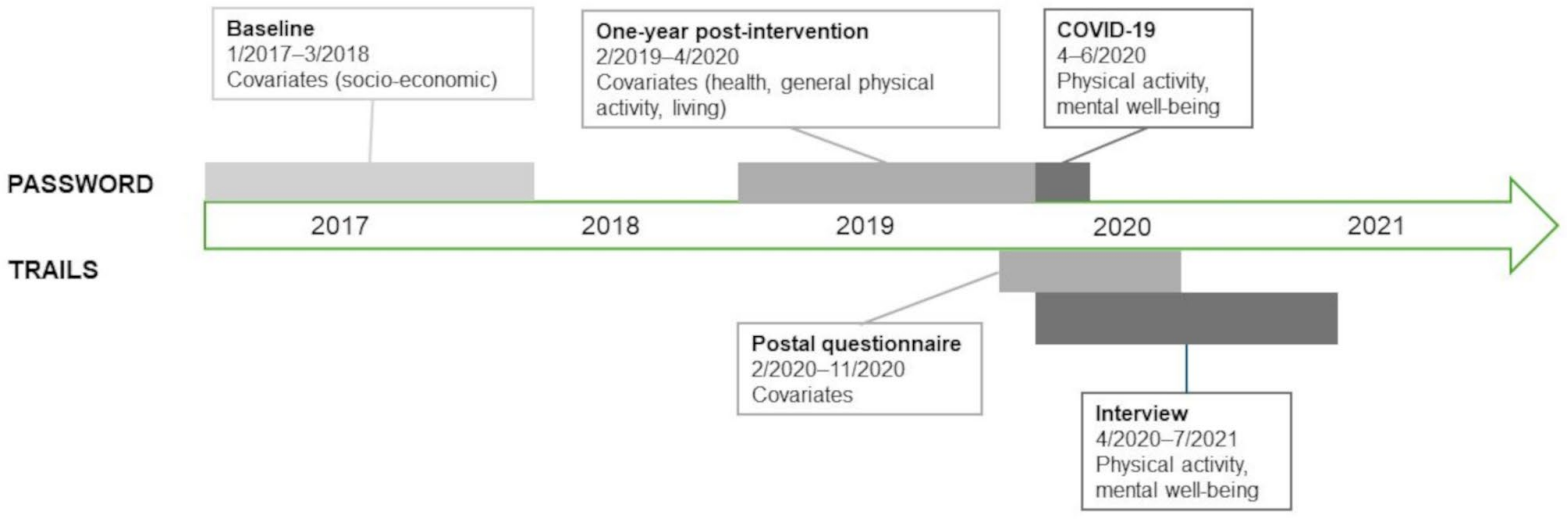
Timeline of the TRAILS and PASSWORD studies

Data for the older cohort were drawn from the PASSWORD study (“Promoting safe walking among older people”, ISRCTN52388040), which was a 12-month randomized controlled trial with a one-year post-intervention follow-up, and an extended follow-up during COVID-19 [13, 33, 34]. Initially, a random sample of 3862 men and women aged 70–85 years living in the City of Jyväskylä, was drawn from the national population registry. After screening telephone calls and laboratory visits, 314 physically inactive, community-dwelling men and women were recruited between January 2017 and March 2018 and randomized to receive either physical training or physical and cognitive training. A postal questionnaire considering PA and MWB during COVID-19 restrictions was mailed to the participants who had not withdrawn from the study (*N* = 288) at the end of April 2020 and the data collection ended in June 2020. The final sample of the present study includes 272 participants (72–88 years, 87% of the initially recruited sample) who responded and had valid data on at least one PA and one MWB question. Previously published attrition analyses showed that those who did not respond to the COVID-19 questionnaire were older, less active, and had poorer lower extremity functioning at the baseline than those who responded to the COVID-19 questionnaire [13]. The timeline of the data collection of the two research projects is presented in Fig. 1.

The TRAILS and PASSWORD studies were conducted in accordance with the Declaration of Helsinki. The PASSWORD study was prospectively registered in the ISCRTN registry (ISRCTN52388040, registration date: 20/01/2017). The TRAILS study protocol was approved by the Ethical Committee of the University of Jyväskylä (December 13, 2019), and the PASSWORD study protocol by the Ethics Committee of the Central Finland Health Care District (14/12/2016, ref: 11/2016; 24/4/2020, ref: 11U/2016). All participants gave their written informed consent.

### Measures

#### Physical activity during the COVID-19 restrictions

Physical activity was assessed by a questionnaire conducted and utilized in several research projects at the Faculty of Sport and Health Sciences, University of Jyväskylä, Finland, to assess the level of and changes in PA during the COVID-19 pandemic [15]. Participants of the younger cohort were interviewed about their PA during COVID-19 restrictions, whereas participants of the older cohort completed the same questionnaire self-administered on paper.

*The frequency of PA* during COVID-19 restrictions was assessed with the question: “How often do you engage in PA or exercise in your leisure time during the present COVID-19 emergency?”. The response scale was: (1) not at all, (2) less than once a month, (3) 1–2 times a month, (4) approximately once a week, (5) 2–3 times a week, (6) 4–5 times a week, and (7) practically every day.

*Perceived changes in PA* during COVID-19 restrictions were assessed with the question “Have you changed your PA or exercise behavior during the COVID-19 pandemic?” The response options were (1) No; (2) Yes, increased a lot; (3) Yes, increased a little; (4) Yes, reduced a little; and (5) Yes, reduced a lot. For further analysis, the response options were recoded as “no change” (category 1), “increased” (2–3), and “reduced” (4–5).

#### Mental well-being

*Positive and negative affect* were assessed by the Finnish version of the International Positive and Negative Affect Schedule Short Form (I-PANAS-SF) [35], in which participants were asked to think about how they usually feel. Ten items, five for positive (e.g., active, determined) and five for negative affect (e.g., afraid, nervous), were presented and rated on a five-point Likert scale, the response options ranging from 1) does not describe me at all to 5) describes me very well. Mean scores were calculated for each subscale. The internal consistency of both positive and negative affect scales was good in the present study. Cronbach’s alphas for positive affect were 0.82 and 0.84 and for negative affect 0.70 and 0.78 in the younger and older cohorts, respectively.

*Depressive symptoms* were assessed with different questionnaires in the two studies. The depression scale of the General Behavior Inventory (GBI) [36] was used to assess depressive symptoms in the younger cohort. The questionnaire includes 16 items, e.g. “Have you become sad, depressed, or irritable for several days or more without really understanding why?”, which are rated on a four-point Likert scale ranging from 1) never to 4) very often. The mean score of the 16 items, the potential range being 1–4, was calculated and used as the outcome variable. Cronbach’s alpha for the measure was 0.91. In the older cohort, the 15-item short form of the Geriatric Depression Scale (GDS-15) [37] was used. The GDS-15 consists of 15 questions with the response options yes/no, e.g., “Are you basically satisfied with your life?” (“no” is scored as 1 point and “yes” as 0 points) and “Do you feel that your life is empty?” (“yes” is scored as 1 point and “no” as 0 points), and a sum score (potential range 0–15) is calculated. For both instruments, a higher score indicates higher depressive symptomology.

In the younger cohort, both MWB questionnaires were completed in face-to-face or online interviews. Participants who were interviewed online received the questionnaires in advance by post. Participants of the older cohort responded to both questionnaires self-administered. The participants who scored 10 points or higher in the GDS-15 were contacted by phone by the study nurse.

#### Covariates

Socio-economic and health-related covariates were chosen based on previous research and data availability in both studies. In the younger cohort, participants were coded as women or men based on the JYLS data collected at the beginning of the longitudinal study in 1968. In the older cohort, sex and date of birth were drawn from the population registry, and age during the COVID-19 outbreak was calculated from the date of birth and the response date of the COVID-19 questionnaire. Of note, all participants in the younger cohort were about the same age, and the exact age was not calculated. Other socio-economic characteristics were drawn from self-reported questionnaires. They included the following: education (university degree vs. no university degree), occupational status (upper white-collar worker vs. lower white-collar worker vs. blue-collar worker), and living with a spouse (yes vs. no). Participants also rated their perceived health (very good/good vs. average/poor/very poor). Data for the younger cohort was collected from the Life Situation questionnaire. They reported their latest occupational title and new education after the previous data collection at the age of 50. Information was complemented from previous data collection phases of the JYLS study [31]. Participants in the older cohort reported their highest education and main or longest-lasting occupation at baseline, and missing data were complemented during following data collection phases when necessary. Data on their perceived health and living situation were collected from the one-year post-intervention follow-up questionnaire.

A single question (“Which of the following descriptions best corresponds to your physical activity at the moment?”) was used to assess the participant’s general PA level [38]. The response options were: (0) I do not move more than is necessary in my daily chores, (1) I go for casual walks and engage in light outdoor recreation 1–2 times a week, (2) I go for casual walks and engage in light outdoor recreation several times a week, (3) I engage 1–2 times a week in brisk physical activity (e.g. yard work, walking, cycling) to the point of perspiring and some degree of breathlessness, (4) I engage several times a week (3–5) in brisk physical activity (e.g. yard work, walking, cycling) to the point of perspiring and some degree of breathlessness, (5) I do keep-fit exercises several times a week in a way that causes rather strong shortness of breath and sweating during the activity, and (6) I participate in competitive sports and maintain my fitness through regular training. The variable was recoded into low (categories 0–1), medium (2–3), and high (4–6) per a previous study [39]. Participants in the younger cohort responded to the question in the interview and were asked to think about their PA level in general, whereas the older participants were asked about their current PA level in the one-year post-intervention follow-up questionnaire.

In the younger cohort, the time between responding to the questionnaire and attending the interview varied from one week to 13 months (median two months) in those participants (*N* = 95) who had marked the response date in the questionnaire. In the older cohort, the time between the one-year post-intervention follow-up questionnaire and the extended follow-up during the COVID-19 restrictions varied from two weeks to 16 months (median six months) depending on the recruitment and response dates

### Statistical analysis

Participant characteristics are summarized as means and standard deviations (SD) for continuous variables and frequencies (n) and percentages (%) for categorical variables. The mutual relationships between positive affect, negative affect, and depressive symptoms were assessed with bivariate Pearson’s correlation coefficients.

The associations of MWB indicators with the frequency of PA during COVID-19 restrictions were analyzed with linear regression models. The frequency of PA was set as the outcome variable. First, each MWB variable (positive affect, negative affect, depressive symptoms) was tested as a predictor each in its own model, adjusted only for sex and, in the older cohort, age (Model 1). Second, all MWB indicators were included as predictors in the same model, adjusted only for sex and, in the older cohort, age (Model 2). In the final, fully adjusted model (Model 3), potentially confounding socio-economic and health-related covariates (education, occupational status, living with a spouse, self-rated health, and general PA level) were added to Model 2. The results from the linear regression models are presented as unstandardized beta coefficients (ß) and 95% confidence intervals (CI).

The associations of MWB indicators with changes in PA were analyzed with multinomial logistic regression models. The change in PA was set as the outcome, and “no change” was selected as the reference category. Predictors and covariates were added to Models 1, 2, and 3 in a similar order as in the linear regression models described above. The results are presented as odds ratios (OR) and 95% CIs.

The assumptions of linear regression analyses were checked based on visual inspection and VIF values. Visual inspection of residual plots indicated homoskedasticity and normality. All VIF values were < 2. The linearity assumption of the multinomial logistic regression analyses was tested with Box-Tidwell tests. The assumption was met for all MWB outcomes in all models in the older cohort (*p* > 0.05). In the younger cohort, the assumption was met in all cases except that the interaction term of depressive symptoms with its natural log predicting reduced PA was statistically significant (*p* = 0.020) in Model 3.

The amount of missing data varied across variables. Regarding the mental well-being indicators, the mean values of positive and negative affect and GBI were calculated for the responses given by the participants. That is, the missing values were imputed by the mean value of other responses. For the older cohort, participants with incomplete GDS-15 questionnaires were excluded from the analysis. In the younger cohort, one participant (1%) had missing data on depressive symptoms. In the older cohort, one participant (0%) had missing data in PA frequency, two (1%) in depressive symptoms, 12 (4%) in negative affect, and 13 (4%) in positive affect. There were no missing data in any covariate. We performed complete-case analyses, in which participants with missing values for the outcome variable (PA frequency or change in PA) or any predictor variable included in the model in question were excluded. The sample size for the different models thus varied from 161 to 162 in the younger and from 255 to 270 in the older cohort, respectively. All analyses were performed with SPSS 28.0 (IBM Corp, Armonk, NY), and the statistical significance level (alpha) was set at 0.05. The a priori sample size calculations for the initial sample sizes have been described in previous publications [31, 33].

## Results

### Descriptive characteristics

Descriptive characteristics are summarized in Table 1 for the younger and older cohorts separately. The proportion of female participants was 56% and 60% in the younger and older cohorts, respectively. The mean age of the older cohort was 77 years during the COVID-19 outbreak. In the younger and older cohorts, 50% and 48% of the participants reported engaging in PA or exercise at least four times per week during COVID-19, respectively. In both cohorts, approximately one in five reported increased PA, and two in five reduced PA during COVID-19. Participants scored on average higher points in positive affect than in negative affect, the median scores being 3.8 and 3.6 in the positive affect scale in the younger and older cohorts, respectively, and 1.2 in the negative affect scale in both cohorts. The median scores in the depression scales were 1.2 points on a scale from 1 to 4 in the younger cohort and 2 points on a scale from 0 to 15 in the older cohort, both indicating low depressive symptomology. In both cohorts, positive affect had a weak but statistically significant negative association with negative affect and a moderate negative association with depressive symptoms (*r* = -0.16 to -0.18, *p* < 0.05, and *r* = -0.35 to -0.45, *p* < 0.001, respectively), while negative affect and depressive symptoms had a moderate positive correlation (*r* = 0.46 to 0.50, *p* < 0.001).

**Table 1:** Participant characteristics by study cohort, mean (SD) or frequency (%)

	Younger cohort *N* = 162	Older cohort *N* = 272
Age, years, mean (SD)	NA	77.0 (3.8)
Women, *n* (%)	91 (56)	162 (60)
Good/very good self-rated health, *n* (%)	108 (67)	150 (55)
Living with a spouse, *n* (%)	123 (76)	161 (59)
University degree, *n* (%)	29 (18)	58 (21)
Occupational status, *n* (%)		
Upper white-collar worker	55 (34)	87 (32)
Lower white-collar worker	67 (41)	82 (30)
Blue-collar worker	40 (25)	103 (38)
General PA level, *n* (%)		
High	77 (48)	80 (29)
Medium	65 (40)	122 (45)
Low	20 (12)	70 (26)
PA frequency during COVID-19, mean (SD), range 1–7 ^a^	5 (2)	5 (1)
Change in PA during COVID-19, *n* (%)		
No change	72 (44)	120 (44)
Increased	28 (17)	50 (18)
Reduced	62 (38)	102 (38)
Positive affect, mean (SD) ^b^	3.7 (0.6)	3.5 (0.7)
Negative affect, mean (SD) ^c^	1.4 (0.4)	1.4 (0.5)
Depressive symptoms, mean (SD)		
GBI, mean score, range 1–4 ^d^	1.5 (0.4)	
GDS-15, total score, range 0–15 ^e^		2.8 (2.6)

Note

^a^ Missing *N* = 1 in the older cohort

^b^ Missing *N* = 13 in the older cohort

^c^ Missing *N* = 12 in the older cohort

^d^ Missing *N* = 1 in the younger cohort

^e^ Missing *N* = 2 in the older cohort

Abbreviations: SD = standard deviation; PA = physical activity; GBI = General Behavior Inventory; GDS-15 = Geriatric Depression Scale

### Associations of mental well-being with the frequency of and changes in PA during the COVID-19 restrictions

Associations of positive affect, negative affect, and depressive symptoms with the frequency of PA during the COVID-19 restrictions are presented in Table 2. Higher positive affect was associated with higher, and depressive symptoms with lower PA frequency in both cohorts, when investigated as the single MWB indicator in the model and only adjusted for sex and age (Model 1). In the younger cohort, these associations did not remain statistically significant when all MWB indicators were investigated in the same model and further adjusted for socioeconomic and health-related factors (Models 2 and 3). In the older cohort, these associations remained relatively stable when all MWB indicators were included in the same model (Model 2), but only the association between higher depressive symptomology and lower PA frequency remained significant when socioeconomic and health-related covariates were added to the model (Model 3). Negative affect was not statistically significantly associated with the frequency of PA in either cohort in any model.

**Table 2:** The associations between mental well-being and the frequency of physical activity during the COVID-19 restrictions

	Younger cohort		Older cohort	
	ß (95% CI)	*p*	ß (95% CI)	*p*
**Model 1**				
Positive affect	**0.489 (0.123**, **0.855)**	**0.009**	**0.550 (0.289**, **0.812)**	**< 0.001**
Negative affect	-0.504 (-1.094, 0.087)	0.094	0.036 (-0.314, 0.387)	0.838
Depressive symptoms*	**-0.876 (-1.472**, **-0.281)**	**0.004**	**-0.105 (-0.171**, **-0.039)**	**0.002**
**Model 2**				
Positive affect	0.306 (-0.091, 0.702)	0.129	**0.420 (0.124**, **0.717)**	**0.005**
Negative affect	-0.105 (-0.753, 0.543)	0.749	0.359 (-0.034, 0.751)	0.073
Depressive symptoms*	-0.632 (-1.340, 0.076)	0.080	**-0.100 (-0.188**, **-0.012)**	**0.026**
**Model 3**				
Positive affect	0.117 (-0.248, 0.482)	0.528	0.175 (-0.121, 0.472)	0.245
Negative affect	-0.104 (-0.682, 0.474)	0.724	0.264 (-0.106, 0.635)	0.161
Depressive symptoms*	-0.621 (-1.271, 0.026)	0.061	**-0.097 (-0.182**, **-0.013)**	**0.024**

Note

Bold font indicates statistical significance (*p* < 0.05)

Unstandardized beta coefficients, their 95% confidence intervals and *p*-values from linear regression analysis

*The beta coefficients for depressive symptoms are not comparable across the cohorts due to different scoring of the instruments (younger cohort: score range 1–4; older cohort: score range 0–15)

**Model 1** includes only one mental well-being indicator at the time, adjusted for sex and, in the older cohort, age

**Model 2** includes all three mental well-being indicators, adjusted for sex, and, in the older cohort, age

**Model 3** includes all three mental well-being indicators, adjusted for sex, occupational status, education, living with a spouse, self-reported health, general physical activity level, and, in the older cohort, age

Of the socioeconomic and health-related factors, participants’ higher general PA level was associated with the frequency of PA in both the younger and the older cohort (ß = 1.047, *p* < 0.001, and ß = 0.713, *p* < 0.001, respectively). That is, those who belonged to the more active groups before the pandemic also reported higher PA frequency during the COVID-19 restrictions than those who reported low general PA levels. All models are presented in detail in Additional File 1.

Associations of positive affect, negative affect, and depressive symptoms with perceived changes in PA during the COVID-19 restrictions are presented in Table 3. In the younger cohort, each one-point increase in the positive affect score was associated with a 208% higher likelihood of increased PA, when investigated separately from the negative MWB indicators and only adjusted for sex (Model 1). This association remained significant when negative MWB indicators and socioeconomic and health-related factors were included in the model (Models 2 and 3). No associations were observed between positive affect and the likelihood of reduced PA. Negative affect or depressive symptoms were not associated with changes in PA in the younger cohort.

**Table 3:** The associations between mental well-being and changes in physical activity during COVID-19 restrictions

	Younger cohort				Older cohort			
	Increased vs. no change		Reduced vs. no change		Increased vs. no change		Reduced vs. no change	
	OR (95% CI)	*p*	OR (95% CI)	*p*	OR (95% CI)	*p*	OR (95% CI)	*p*
**Model 1**								
Positive affect	**3.08 (1.34**, **7.09)**	**0.008**	1.32 (0.74, 2.35)	0.343	1.49 (0.86, 2.60)	0.156	0.71 (0.46, 1.09)	0.117
Negative affect	0.82 (0.25, 2.68)	0.744	1.12 (0.46, 2.76)	0.802	1.17 (0.55, 2.47)	0.686	**2.26 (1.26**, **4.03)**	**0.006**
Depressive symptoms*	0.93 (0.27, 3.21)	0.905	1.42 (0.56, 3.63)	0.462	1.02 (0.85, 1.21)	0.847	**1.32 (1.15**, **1.50)**	**< 0.001**
**Model 2**								
Positive affect	**3.68 (1.51**, **8.99)**	**0.004**	1.60 (0.84, 3.05)	0.151	1.73 (0.93, 3.23)	0.085	1.04 (0.62, 1.74)	0.879
Negative affect	0.87 (0.23, 3.29)	0.833	0.94 (0.33, 2.65)	0.909	1.10 (0.48, 2.55)	0.816	1.47 (0.76, 2.86)	0.257
Depressive symptoms*	2.15 (0.48, 9.59)	0.314	2.00 (0.64, 6.29)	0.236	1.09 (0.89, 1.35)	0.404	**1.28 (1.08**, **1.51)**	**0.004**
**Model 3**								
Positive affect	**4.28 (1.53**, **11.99)**	**0.006**	1.46 (0.73, 2.90)	0.281	1.82 (0.93, 3.55)	0.080	1.10 (0.64, 1.92)	0.725
Negative affect	0.80 (0.21, 3.06)	0.745	0.93 (0.30, 2.94)	0.907	1.13 (0.48, 2.68)	0.784	1.63 (0.81, 3.27)	0.167
Depressive symptoms*	1.45 (0.26, 8.00)	0.668	2.96 (0.85, 10.29)	0.088	1.07 (0.85, 1.33)	0.577	**1.26 (1.06**, **1.50)**	**0.010**

Note

Odds ratios (OR), their 95% confidence intervals (CI) and *p*-values from binary logistic regression analysis

*The odds ratios for depressive symptoms are not comparable across the cohorts due to different scoring of the instruments (younger cohort: score range 1–4; older cohort: score range 0–15)

**Model 1** includes only one mental well-being indicator at the time, adjusted for sex and, in the older cohort, age

**Model 2** includes all three mental well-being indicators, adjusted for sex, and, in the older cohort, age

**Model 3** includes all three mental well-being indicators, adjusted for sex, occupational status, education, living with a spouse, self-reported health, general physical activity level, and, in the older cohort, age

In the older cohort, each one-point increase in negative affect and depressive symptoms was associated with a 126% and 32% higher likelihood of reduced PA during COVID-19 restrictions, respectively, when they were investigated as single MWB indicators in the model and only adjusted sex and age (Model 1). Only the association between depressive symptoms and reduced PA remained statistically significant when all MWB indicators were included in the same model and further adjusted for socioeconomic and health-related covariates (Models 2 and 3). No associations were observed between negative affect or depressive symptoms and the likelihood of reporting increased PA or between positive affect and changes in PA in the older cohort. All models are shown in detail in Additional File 2.

## Discussion

In this cross-sectional analysis, we found that positive affect was associated with a higher likelihood of reporting increased physical activity during the COVID-19 pandemic in sixty-year-olds independent of negative mental well-being, and socioeconomic and health-related factors. Higher depressive symptoms, in turn, were associated with lower physical activity frequency and a higher likelihood of reporting reduced physical activity during the pandemic in those aged 72 and older, independent of positive and negative affect and socioeconomic and health-related factors.

The results of the present study complement the existing literature indicating that positive mental well-being may promote healthy behavior [21–23, 40] by providing novel information on the associations of positive affect with PA during the COVID-19 restrictions in people aged 60 years and older, in whom research has so far been sparse. Positive affect is suggested to facilitate motivation and make people more resistant to stress, which is linked to healthier behaviors [20, 41], and may support the adaption of one’s PA behavior also during stressful situations such as during a global pandemic. In the present study, higher positive affect was associated with higher PA frequency during the COVID-19 restrictions across late adulthood in the initial models, in which positive affect was the single MWB indicator. However, these associations did not remain significant when both negative MWB and socioeconomic and health-related factors were accounted for. These findings suggest that other characteristics, such as the presence of depressive symptoms and socioeconomic status, may partly explain the relationship between positive affect and PA. Especially the general PA level was strongly associated with the frequency of PA during COVID-19 in both cohorts. That is, those who had been active before the pandemic remained active during the social distancing restrictions.

Interestingly, the associations of positive affect with self-reported changes in PA during the COVID-19 restrictions differed between the younger and older cohorts. In the cohort of 61-year-olds, a significant association was observed between higher positive affect and a higher likelihood of reporting increased PA during the pandemic, independent of negative mental well-being indicators and confounding factors. In contrast, statistically significant associations were not found in the 72-88-year-olds. These findings are partly contrasting to the few previous studies investigating the relationships between positive affect and PA in late adulthood. Chen and colleagues [40] found that the relationship between higher positive MWB and higher PA strengthened with increasing age in Swedish adults aged 60 years and older. In contrast, Holahan and colleagues [42] did not find significant differences in the association of positive affect with PA between US women aged 45 to 60 years and those aged 61 to 75 years. The discrepancies between the present study and other studies may at least partly be explained by that the associations between MWB and PA may change during challenging circumstances, such as during the COVID-19 social distancing restrictions.

Our findings on the inverse relationships between depressive symptoms and PA are in line with the existing literature conducted in adult populations during the COVID-19 pandemic [16] and provide novel information on these associations in people aged 70 years and older, in whom research is lacking. In the present study, higher depressive symptoms were associated with a lower frequency of PA across adulthood, when it was investigated as the single MWB indicator in the model. However, when positive and negative affect and potential confounders were included in the model, the association remained statistically significant in the older cohort only. Additionally, higher depressive symptoms were associated with a higher likelihood of reduced PA in the older cohort independent of positive and negative affect and other factors, indicating that depressive symptoms are an independent risk factor for low PA during the COVID-19 restrictions in older adults. Depression may link to poorer health behaviors both via lower motivation and energy but also have direct physiological links [9], which may explain why depressive symptoms were the only mental well-being indicator that was a significant predictor of PA behavior in the older cohort in the fully adjusted model.

Of note, the data from the older cohort were almost completely collected when they were obliged to self-quarantine. Quarantine is known to reduce MWB [43] and approximately one in four Finnish people of the same age isolated themselves totally [44]. Furthermore, many public sports facilities and group activities that are popular among older adults were closed. The younger cohort was not required to self-quarantine even at the very beginning of the COVID-19 outbreak. Additionally, the data collection period of the younger cohort was longer than that of the older cohort, and the general restrictions were not as strict throughout the whole data collection period. Therefore, depressive feelings and external barriers may have influenced physical activity behavior and overcome the potentially beneficial role of positive affect to a greater extent in the older compared to the younger cohort since their lives were restricted more.

In accordance with previous studies [17, 21–23, 40], negative affect was not related to PA in either cohort when all three MWB indicators were inspected together. There is, however, some indicative evidence that an inverse association between negative affect and PA may be found in older age. In a Spanish cohort, a negative association was found between negative affect and PA in people aged 65 years and older, but not among those aged 50–64 years [45]. This is in line with our finding that as a single predictor negative affect was related to a higher likelihood of reduced PA in the older but not in the younger cohort. However, of the negative MWB indicators, only depressive symptoms were associated with PA when all MWB indicators were inspected in the same model. This may be explained by that depressive symptomology covers both depressed mood and diminished interests, which are related to high negative and low positive affect, respectively, but also other symptoms such as impaired cognitive function and disturbed sleep [5, 24, 46]. Depressive symptoms are thus a more comprehensive risk factor for low PA than negative affect alone. However, the independent relationships between positive and negative MWB indicators and PA deserve further research utilizing larger samples including participants from a wider age range, longitudinal designs, and both self-reported and device-based assessment of PA.

## Limitations and strengths

This study has its limitations. First, this was an exploratory post-hoc analysis of two larger studies. That is, the sample sizes and methodologies were not fully optimized for the present research questions. Data on socio-economic and health-related characteristics were collected somewhat earlier than data on PA. One limitation regarding the self-reported changes in PA is that in we don’t know whether the perceived changes in PA volume were due to changes in intensity, frequency, and/or duration of PA. There were also some differences in the methodologies utilized in the two research projects. For example, different depression scales were used and the results regarding depressive symptoms are thus not fully comparable between the two cohorts. Additionally, the data collection period of the younger cohort was notably longer than that of the older cohort. It may be that the results of the younger cohort would have been different, if all data had been collected during the strictest restrictions between April and June 2020. An important limitation of the present study is its cross-sectional nature, i.e., we cannot draw any conclusions on the causality of the relationships between MWB and PA. Unfortunately, we did not have timely information on risk factors for severe COVID-19 in the older cohort. In this age group, health and functioning may change rapidly and the health and functioning of one’s spouse may be of importance, which we could not account for. Furthermore, although recruited from a population-based random sample, older adults who agreed to participate in a year-long exercise intervention and underwent strict behavioral and clinical screening (i.e., they had to be initially inactive but healthy enough to participate in an intensive exercise program), may not have been representative of their age cohort. After participating in a structured exercise program, they also may have had better prerequisites to adapt their physical activity to the COVID-19 restrictions than older adults in general. Lastly, the relatively small sample sizes, especially in the younger cohort, may have led to a lack of statistical power in the fully adjusted model, and larger sample sizes may be needed to reveal the independent associations of different aspects of mental well-being with physical activity outcomes in these populations.

Despite the limitations, this study also has several strengths. First, we utilized data from two larger population-based studies, while most studies conducted during the COVID-19 pandemic have utilized convenience sampling potentially leading to sampling bias. The younger cohort was still relatively well representative of their age cohort in Finland, although women were overrepresented compared to the initial sample [32]. The participants of the older cohort, in turn, were relatively similar to their age cohort living in the same area. For example, they reported living with a spouse, good to very good health, and high general PA levels approximately as often as 75-year-olds living in the same area, who participated in a large cohort study [47, 48]. Second, it was possible to include data from these two cohorts of different ages since the studies used comparable methodology despite some minor differences. We could investigate potential differences in the associations between MWB and PA during the COVID-19 restrictions between two age-cohorts in late adulthood at high risk of reduced PA and MWB. Furthermore, the cohorts were even surprisingly similar regarding affectivity, PA during the COVID-19 restrictions, and several socioeconomic characteristics. Similarly, the mutual associations between the three MWB indicators were very similar in both cohorts. Investigating aspects of positive and negative MWB together was an additional benefit in the present study and adds to the existing literature. The generalizability of the results of the present study can be assumed to be relatively good in these age cohorts of Finnish older adults. It must, however, be borne in mind that although the initial sample was drawn from the population registry, the older cohort had attended a year-long physical activity intervention including home-based exercise. Therefore, they may have been more capable of adapting their physical activity behavior during COVID-19 than their peers.

## Conclusions

In conclusion, the present study provides important new information on the independent associations of positive and negative aspects of mental well-being with physical activity during COVID-19 restrictions in people aged 60 years and older, in whom population-based research is lacking during such exceptional circumstances. Positive affect was associated with a higher likelihood of reporting increased physical activity in sixty-year-olds independent of negative mental well-being indicators, and socioeconomic and health-related factors. Higher depressive symptomology, in turn, was independently associated with a higher likelihood of reporting reduced physical activity in those aged 72 and older, and with lower physical activity frequency across late adulthood. These findings highlight that positive affect and depressive symptoms may have independent associations with physical activity behavior when one’s daily routines are restricted, but the relationships may be different in different phases of late adulthood. Furthermore, the findings of the present study suggest that depressive symptoms may be a better indicator of negative mental well-being than negative affect when identifying older adults at risk of low physical activity. Altogether, during such exceptional circumstances as a global pandemic, enhancing positive affect may support a physically active lifestyle at the onset of late adulthood, whereas counteracting depressive symptoms may be more important in older age or under stricter restrictions.

## Electronic supplementary material

Below is the link to the electronic supplementary material.

Additional file 1: Supplementary Table 1. The associations between mental well-being and the frequency of physical activity during the COVID-19 restrictions

Additional file 2: Supplementary Table 2. The associations between mental well-being and reporting increased physical activity during the COVID-19 restrictions

## Data Availability

The datasets generated and/or analyzed during the current study are not publicly available due to the sensitivity of the data and privacy of the participants. The data analyses that support the findings of the present article are available from the corresponding author upon reasonable request. Pseudonymized datasets are available to external collaborators upon agreement on the terms of data use and publication of results. To request the data of the JYLS/TRAILS study, please contact the Principal Investigator Dr. Katja Kokko (katja.r.kokko@jyu.fi). To request the data of the PASSWORD study, please contact the Principal Investigator, Professor Sarianna Sipilä (Sarianna.sipila@jyu.fi), University of Jyväskylä.
