# Supplementary material for: The associations of positive and negative mental well-being with physical activity during the COVID-19 across late adulthood
Source: BMC Public Health. 2024 Nov 26;24:3288. doi: 10.1186/s12889-024-20803-3 (PMC11600867; doi:10.1186/s12889-024-20803-3)
Supplement: Supplementary file 1 — Additional file 1: Supplementary Table 1. The associations between mental well-being and the frequency of physical activity during the COVID-19 restrictions [file 12889_2024_20803_MOESM1_ESM.docx]

Supplementary Table 1. The associations between mental well-being and the frequency of physical activity during the COVID-19 restrictions.

|  | **Younger cohort** | | | | |  | **Older cohort** | | | | |
| --- | --- | --- | --- | --- | --- | --- | --- | --- | --- | --- | --- |
|  | **ß** | **SE of ß** | **95% CI of ß (lower, upper)** | **p** | **Model statistics** |  | **ß** | **SE of ß** | **95% CI of ß (lower, upper)** | **p** | **Model statistics** |
| **Model 1** |  |  |  |  |  |  |  |  |  |  |  |
| Positive affect | 0.489 | 0.185 | 0.123, 0.855 | 0.009 |  |  | 0.550 | 0.133 | 0.289, 0.812 | <0.001 |  |
| Sex (male) | -0.587 | 0.236 | -1.054, -0.120 | 0.014 |  |  | 0.063 | 0.178 | -0.287, 0.414 | 0.722 |  |
| Age | NA | NA | NA | NA |  |  | -0.029 | 0.023 | -0.074, 0.017 | 0.214 |  |
| F |  |  |  |  | 8.151 |  |  |  |  |  | 6.633 |
| Df |  |  |  |  | 2 |  |  |  |  |  | 3 |
| R^2^ |  |  |  |  | 0.093 |  |  |  |  |  | 0.073 |
| Adjusted R^2^ |  |  |  |  | 0.082 |  |  |  |  |  | 0.062 |
| P for model |  |  |  |  | <0.001 |  |  |  |  |  | <0.001 |
|  |  |  |  |  |  |  |  |  |  |  |  |
| Negative affect | -0.504 | 0.299 | -1.094, 0.087 | 0.094 |  |  | 0.036 | 0.178 | -0.314, 0.387 | 0.838 |  |
| Sex (male) | -0.755 | 0.236 | -1.222, -0.288 | 0.002 |  |  | 0.085 | 0.185 | -0.280, 0.449 | 0.648 |  |
| Age | NA | NA | NA | NA |  |  | -0.036 | 0.024 | -0.084, 0.012 | 0.137 |  |
| F |  |  |  |  | 5.969 |  |  |  |  |  | 0.852 |
| Df |  |  |  |  | 2 |  |  |  |  |  | 3 |
| R^2^ |  |  |  |  | 0.07 |  |  |  |  |  | 0.010 |
| Adjusted R^2^ |  |  |  |  | 0.058 |  |  |  |  |  | -0.002 |
| P for model |  |  |  |  | 0.003 |  |  |  |  |  | 0.457 |
|  |  |  |  |  |  |  |  |  |  |  |  |
| Depressive symptoms* | -0.876 | 0.301 | -1.472, -0.281 | 0.004 |  |  | -0.105 | 0.034 | -0.171, -0.039 | 0.002 |  |
| Sex (male) | -0.826 | 0.233 | -1.289, -0.368 | <0.001 |  |  | -0.022 | 0.177 | -0.370, 0.327 | 0.903 |  |
| Age | NA | NA | NA | NA |  |  | -0.030 | 0.023 | -0.074, 0.015 | 0.189 |  |
| F |  |  |  |  | 9.302 |  |  |  |  |  | 3.904 |
| Df |  |  |  |  | 2 |  |  |  |  |  | 3 |
| R^2^ |  |  |  |  | 0.105 |  |  |  |  |  | 0.042 |
| Adjusted R^2^ |  |  |  |  | 0.094 |  |  |  |  |  | 0.031 |
| P for model |  |  |  |  | <0.001 |  |  |  |  |  | 0.009 |
|  |  |  |  |  |  |  |  |  |  |  |  |
| **Model 2** |  |  |  |  |  |  |  |  |  |  |  |
| Positive affect | 0.306 | 0.201 | -0.091, 0.702 | 0.129 |  |  | 0.420 | 0.151 | 0.124, 0.717 | 0.005 |  |
| Negative affect | -0.105 | 0.328 | (-0.753, 0.543 | 0.749 |  |  | 0.359 | 0.199 | -0.034, 0.751 | 0.073 |  |
| Depressive symptoms* | -0.632 | 0.358 | -1.340, 0.076 | 0.080 |  |  | -0.100 | 0.044 | -0.188, -0.012 | 0.026 |  |
| Sex (male) | -0.733 | 0.242 | -1.212, -0.255 | 0.003 |  |  | 0.010 | 0.182 | -0.349, 0.369 | 0.955 |  |
| Age | NA | NA | NA | NA | NA |  | -0.025 | 0.024 | -0.071, 0.022 | 0.296 |  |
|  |  |  |  |  |  |  |  |  |  |  |  |
| Chi-square |  |  |  |  | 5.280 |  |  |  |  |  | 5.076 |
| Df |  |  |  |  | 4 |  |  |  |  |  | 5 |
| R^2^ |  |  |  |  | 0.119 |  |  |  |  |  | 0.092 |
| Adjusted R^2^ |  |  |  |  | 0.097 |  |  |  |  |  | 0.074 |
| P for model |  |  |  |  | <0.001 |  |  |  |  |  | <0.001 |
|  |  |  |  |  |  |  |  |  |  |  |  |
| **Model 3** |  |  |  |  |  |  |  |  |  |  |  |
| Positive affect | 0.117 | 0.185 | -0.248, 0.482 | 0.528 |  |  | 0.175 | 0.151 | -0.121, 0.472 | 0.245 |  |
| Negative affect | -0.104 | 0.293 | -0.682, 0.474 | 0.724 |  |  | 0.264 | 0.188 | -0.106, 0.635 | 0.161 |  |
| Depressive symptoms* | -0.621 | 0.329 | -1.271, 0.026 | 0.061 |  |  | -0.097 | 0.043 | -0.182, -0.013 | 0.024 |  |
| Sex (male) | -0.671 | 0.220 | -1.106, -0.236 | 0.003 |  |  | -.0.033 | 0.181 | -0.391, 0.324 | 0.854 |  |
| Age | NA | NA | NA | NA |  |  | -0.010 | 0.023 | -0.055, 0.034 | 0.648 |  |
| Living with spouse | -0.356 | 0.248 | -0.845, 0.133 | 0.153 |  |  | -0.132 | 0.181 | -0.488, 0.224 | 0.465 |  |
| Good/very good health | -0.208 | 0.231 | -0.663, 0.248 | 0.369 |  |  | -0.045 | 0.194 | -0.428, 0.337 | 0.816 |  |
| University degree | 0.121 | 0.307 | -0.486, 0.728 | 0.694 |  |  | 0.111 | 0.248 | -0.379, 0.600 | 0.657 |  |
| Occupation | -0.067 | 0.159 | -0.381, 0.247 | 0.674 |  |  | 0.042 | 0.124 | -0.203, 0.287 | 0.734 |  |
| General PA | 1.047 | 0.156 | 0.740, 1.135 | <0.001 |  |  | 0.713 | 0.125 | 0.467, 0.959 | <0.001 |  |
| F |  |  |  |  | 8.221 |  |  |  |  |  | 6.858 |
| Df |  |  |  |  | 9 |  |  |  |  |  | 10 |
| R^2^ |  |  |  |  | 0.329 |  |  |  |  |  | 0.219 |
| Adjusted R^2^ |  |  |  |  | 0.289 |  |  |  |  |  | 0.187 |
| P for model |  |  |  |  | <0.001 |  |  |  |  |  | <0.001 |

Note.

*The beta coefficients for depressive symptoms are not comparable across the cohorts due to different scoring of the instruments (younger cohort: score range 1-4; older cohort: score range 0-15).

**Model 1** includes only one mental well-being indicator at the time, adjusted for sex and, in the older cohort, age.
**Model 2** includes all three mental well-being indicators, adjusted for sex, and, in the older cohort, age.
**Model 3** includes all three mental well-being indicators, adjusted for sex, occupational status, education, living with a spouse, self-reported health, general physical activity level, and, in the older cohort, age.

Abbreviations: ß = unstandardized beta coefficient; SE = standard error; CI = confidence interval; Df = degrees of freedom.
