# Supplementary material for: The associations of positive and negative mental well-being with physical activity during the COVID-19 across late adulthood
Source: BMC Public Health. 2024 Nov 26;24:3288. doi: 10.1186/s12889-024-20803-3 (PMC11600867; doi:10.1186/s12889-024-20803-3)
Supplement: Supplementary file 2 — Additional file 2: Supplementary Table 2. The associations between mental well-being and reporting increased physical activity during the COVID-19 restrictions [file 12889_2024_20803_MOESM2_ESM.docx]

Supplementary Table 2. The associations between mental well-being and changes in physical activity during the COVID-19 restrictions.

|  | **Younger cohort** | | | | |  | **Older cohort** | | | | |
| --- | --- | --- | --- | --- | --- | --- | --- | --- | --- | --- | --- |
|  | **Increased vs. no change** | |  | **Reduced vs. no change** | |  | **Increased vs. no change** | |  | **Reduced vs. no change** | |
|  | **OR (95% CI)** | **p** |  | **OR (95% CI)** | **p** |  | **OR (95% CI)** | **p** |  | **OR (95% CI)** | **p** |
| **Model 1** |  |  |  |  |  |  |  |  |  |  |  |
| Positive affect | **3.08 (1.34, 7.09)** | **0.008** |  | 1.32 (0.74, 2.35) | 0.343 |  | 1.49 (0.86, 2.60) | 0.156 |  | 0.71 (0.46, 1.09) | 0.117 |
| Sex (male) | **0.26 (0.09, 0.70)** | **0.008** |  | **0.32 (0.16, 0.65)** | **0.002** |  | 0.58 (0.29, 1.17) | 0.129 |  | **0.35 (0.19, 0.62)** | **<0.001** |
| Age | **-** | **-** |  | - | - |  | 0.93 (0.84, 1.02) | 0.132 |  | 0.99 (0.92, 1.06) | 0.760 |
| Chi-squared | 24.24 | | | | |  | 23.83 | | | | |
| Df | 4 | | | | |  | 6 | | | | |
| P for model | <0 .001 | | | | |  | <0.001 | | | | |
| AIC | 321.9 | | | | |  | 532-6 | | | | |
| BIC | 340.5 | | | | |  | 550.1 | | | | |
| Nagelkerke R^2^ | 0.07 | | | | |  | 0.10 | | | | |
| Negative affect | 0.82 (0.25, 2.68) | 0.744 |  | 1.12 (0.46, 2.76) | 0.802 |  | 1.17 (0.55, 2.47) | 0.686 |  | **2.26 (1.26, 4.03)** | **0.006** |
| Sex (male) | **0.21 (0.08, 0.56)** | **0.002** |  | **0.31 (0.15, 0.63)** | **0.001** |  | 0.57 (0.28, 1.14) | 0.111 |  | **0.34 (0.19, 0.62)** | **<0.001** |
| Age | - | - |  | - | - |  | 0.92 (0.83, 1.02) | 0.103 |  | 0.99 (0.92, 1.07) | 0.855 |
| Chi-squared | 24.24 |  |  |  |  |  | 27.05 | | | | |
| Df | 4 |  |  |  |  |  | 6 | | | | |
| P for model | < 0.001 |  |  |  |  |  | <0.001 | | | | |
| Nagelkerke R^2^ | 0.09 |  |  |  |  |  | 0.11 | | | | |
| AIC | 329.5 |  |  |  |  |  | 530.0 | | | | |
| BIC | 348.0 |  |  |  |  |  | 558.5 | | | | |
| Depressive symptoms* | 0.93 (0.27, 3.21) | 0.905 |  | 1.42 (0.56, 3.63) | 0.462 |  | 1.02 (0.85, 1.21) | 0.847 |  | **1.32 (1.15, 1.50)** | **<0.001** |
| Sex (male) | **0.22 (0.08, 0.58)** | **0.002** |  | **0.32 (0.16, 0.66)** | **0.002** |  | 0.62 (0.31, 1.21) | 0.161 |  | **0.39 (0.22, 0.71)** | **0.002** |
| Age | - | - |  | - | - |  | 0.93 (0.85, 1.02) | 0.124 |  | 0.98 (0.91, 1.05) | 0.554 |
| Chi-squared | 16.54 | | | | |  | 41.29 | | | | |
| Df | 4 | | | | |  | 6 | | | | |
| P for model | 0.002 | | | | |  | <0.001 | | | | |
| Nagelkerke R^2^ | 0.07 | | | | |  | 536.5 | | | | |
| AIC | 328.0 | | | | |  | 565.4 | | | | |
| BIC | 346.5 | | | | | | 0.16 | | | | |
| **Model 2** |  |  |  |  |  |  |  |  |  |  |  |
| Positive affect | **3.68 (1.51, 8.99)** | **0.004** |  | 1.60 (0.84, 3.05) | 0.151 |  | 1.73 (0.93, 3.23) | 0.085 |  | 1.04 (0.62, 1.74) | 0.879 |
| Negative affect | 0.87 (0.23, 3.29) | 0.833 |  | 0.94 (0.33, 2.65) | 0.909 |  | 1.10 (0.48, 2.55) | 0.816 |  | 1.47 (0.76, 2.86) | 0.257 |
| Depressive symptoms* | 2.15 (0.48, 9.59) | 0.314 |  | 2.00 (0.64, 6.29) | 0.236 |  | 1.09 (0.89, 1.35) | 0.404 |  | **1.28 (1.08, 1.51)** | **0.004** |
| Sex (male) | **0.29 (0.10, 0.80)** | **0.017** |  | **0.36 (0.17, 0.76)** | **0.007** |  | 0.61 (0.30, 1.24) | 0.175 |  | **0.39 (0.21, 0.72)** | **0.002** |
| Age | - | - |  | - | - |  | 0.93 (0.84, 1.02) | 0.121 |  | 0.99 (0.91, 1.07) | 0.718 |
| Chi-squared | 25.77 | | | | |  | 44.13 | | | | |
| Df | 8 | | | | |  | 10 | | | | |
| P for model | 0.001 | | | | |  | <0.001 | | | | |
| Nagelkerke R^2^ | 0.10 | | | | |  | 0.18 | | | | |
| AIC | 326.8 | | | | |  | 513.4 | | | | |
| BIC | 357.6 | | | | |  | 555.9 | | | | |
| **Model 3** |  |  |  |  |  |  |  |  |  |  |  |
| Positive affect | **4.28 (1.53, 11.99)** | **0.006** |  | 1.46 (0.73, 2.90) | 0.281 |  | 1.82 (0.93, 3.55) | 0.080 |  | 1.10 (0.64, 1.92) | 0.725 |
| Negative affect | 0.80 (0.21, 3.06) | 0.745 |  | 0.93 (0.30, 2.94) | 0.907 |  | 1.13 (0.48, 2.68) | 0.784 |  | 1.63 (0.81, 3.27) | 0.167 |
| Depressive symptoms* | 1.45 (0.26, 8.00) | 0.668 |  | 2.96 (0.85, 10.29) | 0.088 |  | 1.07 (0.85, 1.33) | 0.577 |  | **1.26 (1.06, 1.50)** | **0.010** |
| Sex (male) | **0.19 (0.05, 0.75)** | **0.018** |  | **0.37 (0.15, 0.93)** | **0.035** |  | 0.58 (0.26, 1.29) | 0.181 |  | **0.37 (0.18, 0.74)** | **0.005** |
| Age | - | - |  | - | - |  | **0.90 (0.81, 1.00)** | **0.048** |  | 0.97 (0.89, 1.05) | 0.414 |
| Living with spouse | 0.47 (0.15, 1.54) | 0.214 |  | **3.32 (1.19, 9.31)** | **0.022** |  | 0.48 (0.22, 1.02) | 0.056 |  | 0.86 (0.45, 1.67) | 0.661 |
| Good/very good health | 0.50 (0.16, 1.55) | 0.228 |  | 1.03 (0.43, 2.49) | 0.942 |  | 1.09 (0.47, 2.50) |  |  | 0.93 (0.46, 1.87) | 0.830 |
| University degree | 1.77 (0.39, 8.03) | 0.462 |  | 0.44 (0.13, 1.45) | 0.177 |  | 0.42 (0.13, 1.39) | 0.156 |  | 0.77 (0.30, 1.98) | 0.583 |
| Occupation, lower white-collar | 0.27 (0.05, 1.37) | 0.114 |  | 1.52 (0.48, 4.85) | 0.480 |  | 0.90 (0.36, 2.20) | 0.809 |  | 1.23 (0.57, 2.63) | 0.602 |
| Occupation, upper white-collar | 0.18 (0.03, 1.08) | 0.061 |  | 1.45 (0.45, 4.65) | 0.535 |  | 2.10 (0.73, 6.04) | 0.169 |  | 2.24 (0.87, 5.79) | 0.095 |
| General PA, medium | 5.31 (0.43, 65.18) | 0.192 |  | 2.30 (0.63, 8.36) | 0.208 |  | 0.69 (0.26, 1.83) | 0.457 |  | 0.61 (0.28, 1.32) | 0.207 |
| General PA, high | **16.41 (1.32, 203.42** | **0.029** |  | **3.74 (1.00, 13.89)** | **0.049** |  | 0.76 (0.26, 2.20) | 0.611 |  | 0.42 (0.17, 1.06) | 0.066 |
| Chi-squared | 56.54 | | | | |  | 57.60 | | | | |
| Df | 22 | | | | |  | 24 | | | | |
| P for model | <0 .001 | | | | |  | <0.001 | | | | |
| Nagelkerke R^2^ | 0.22 | | | | |  | 0.23 | | | | |
| AIC | 324.0 | | | | |  | 527.9 | | | | |
| BIC | 398.0 | | | | |  | 620.1 | | | | |

Note.

Odds ratios (OR), their 95% confidence intervals (CI) and p-values from binary logistic regression analysis.

*The odds ratios for depressive symptoms are not comparable across the cohorts due to different scoring of the instruments (younger cohort: score range 1-4; older cohort: score range 0-15).

**Model 1** includes only one mental well-being indicator at the time, adjusted for sex and, in the older cohort, age.
**Model 2** includes all three mental well-being indicators, adjusted for sex, and, in the older cohort, age.
**Model 3** includes all three mental well-being indicators, adjusted for sex, occupational status, education, living with a spouse, self-reported health, general physical activity level, and, in the older cohort, age.
